# Crystal structures of pure 3-(4-bromo-2-chloro­phen­yl)-1-(pyridin-4-yl)benzo[4,5]imidazo[1,2-*d*][1,2,4]triazin-4(3*H*)-one and contaminated with 3-(4-bromo­phen­yl)-1-(pyridin-4-yl)benzo[4,5]imidazo[1,2-*d*][1,2,4]triazin-4(3*H*)-one

**DOI:** 10.1107/S2056989017011732

**Published:** 2017-08-15

**Authors:** Kanan Wahedy, Bassam Abu Thaher, Dieter Schollmeyer, Ihab Almasri, Rami Morjan, Basem Qeshta, Hans-Peter Deigner

**Affiliations:** a Faculty of Pharmacy, Department of Pharmaceutical Chemistry, Alazhar University-Gaza, Gaza Strip, Palestinian Territories; b Faculty of Science, Chemistry Department, Islamic University of Gaza Strip, Gaza Strip, Palestinian Territories; c Department of Organic Chemistry, Johannes Gutenberg-University Mainz, Duesbergweg 10-14, 55099 Mainz, Germany; d Hochschule Furtwangen (HFU), Fakultät Medical and Life Sciences, Jakob-Kienzle Strasse 17, 78054 Villingen-Schwenningen, Germany; e Fraunhofer IZI, EXIM Rostock, Perlickstrasse 1, 04103 Leipzig, Germany

**Keywords:** crystal structure, 4-bromo-2-chloro­phen­yl, pyridine, benzo­imidazole, 1,2,4-triazinone

## Abstract

The side product of the cyclo­condensation reaction between ethyl benzimidazole-2-carboxyl­ate and the nitrile imine of the corresponding hydrazonyl chloride, C_20_H_11_BrClN_5_O, crystallized in two crystal forms. Form (**1**) is a co-crystal of the target compound (without any chlorine substituent) and a side product containing a Cl atom in position 2 of the bromo­phenyl group, C_20_H_12_BrN_5_O·0.143C_20_H_11_BrClN_5_O. (**2**) contains the pure side product. The slightly different conformation of the ring systems leads to a different packing of (**1**) and (**2**), but both crystal structures are dominated by π–π inter­actions.

## Chemical context   

Compounds containing a benzimidazole core have been tackled in the area of pharmaceuticals (Karpin’ska *et al.* 2011 ▸; Singh *et al.* (2010 ▸) and therapeutic areas (Biron, 2006 ▸; Pescovitz, 2008 ▸), as well as commercial drugs such as omeprazole (prilosec), pantoprazole (protonix), vermox and mibefradil (Karpińska *et al.*, 2011 ▸). Several benzimidazole-based compounds show anti-cancer activity (Thomas *et al.*, 2007 ▸), and some of them exhibit cytotoxic effects against a panel of human cancer cell lines (Refaat, 2010 ▸). For example, benzimidazole-4,7-diones exhibit cytotoxicity against colon, breast and lung cell lines (Gellis *et al.*, 2008 ▸). The good efficiency of imidazole-based compounds as anti-cancer agents promoted this study of synthesizing a masked benzimidazole in a triazine ring as a new scaffold with potential anti-cancer candidates. The first and the second derivative of this series afforded good crystals and have been published previously (Abu Thaher *et al.*, 2016*a* ▸,*b* ▸). The aim of this study was to prepare 3-(4-bromo­phen­yl)-1-(pyridin-4-yl)benzo[4,5]imid­azo[1,2-*d*][1,2,4]-triazin-4(3*H*)-one.

## Structural commentary   

**Figure 1:**
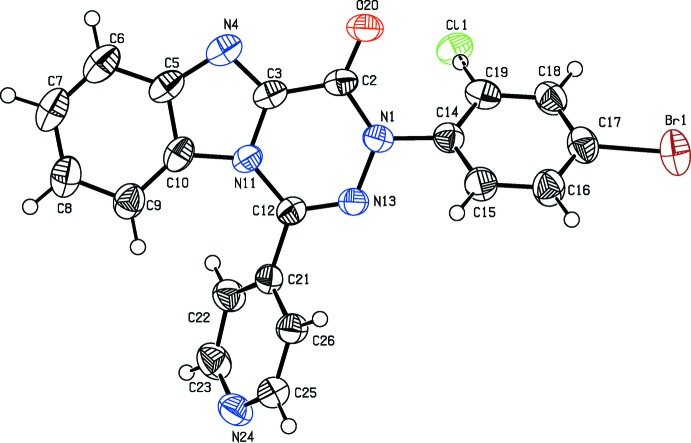
The crystal structure of (**1**), with the atom labelling and displacement ellipsoids drawn at the 50% probability level. The Cl atom has a site-occupation factor of only 1/8.

**Figure 2:**
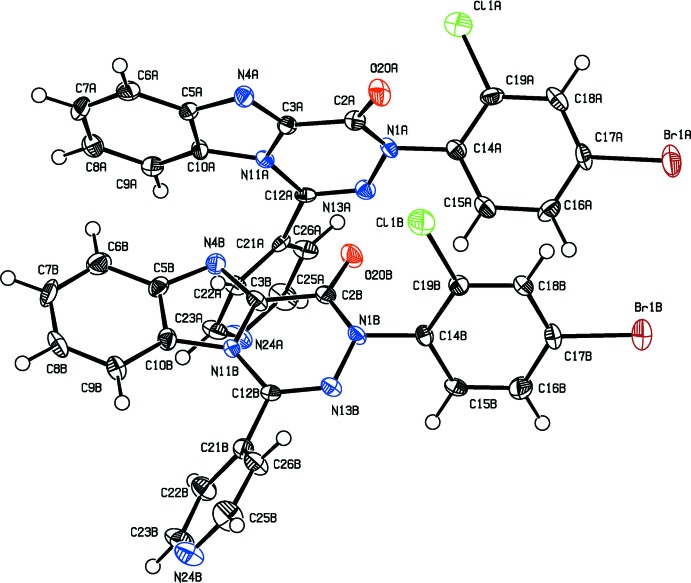
The crystal structure of (**2**) with the atom labelling. Displacement ellipsoids drawn at the 50% probability level. The two independent mol­ecules are labelled with suffixes *A* and *B*.

During crystallization of the product from a bi-solvent mixture of *n*-heptane and EtAc, two types of crystals were obtained. The biggest and highest quality blocks among them, (**1**) (Fig. 1 ▸), were obtained as a co-crystal of the target compound and a side product containing a chlorine atom in position 2 of the bromo­phenyl group. The chlorine atom in it is attached to the bromo­phenyl group as a side product obtained during preparation of the starting material, namely hydrazonoyl chloride, *via* chlorination of the corresponding hydrazone. The quantitative ratio between the side:target product is 1:7. The second type of crystals, (**2**), nice column-like crystals, turned out to contain the pure side product (Fig. 2 ▸). Furthermore, crystals of (**2**) contain two independent mol­ecules (*A* and *B*) in the asymmetric unit. Their geometry is almost identical but different from (**1**) (see Table 1 ▸). The r.m.s. fit of all non-hydrogen atoms from molecule *A* onto *B* is 0.116 Å. The fused 13-membered ring system in (**1**) and (**2**) is nearly planar with an r.m.s. deviation of 0.025 Å in (**1**) and an r.m.s. deviation of 0.100 Å for mol­ecule *A* and 0.089 Å for mol­ecule *B* of (**2**).
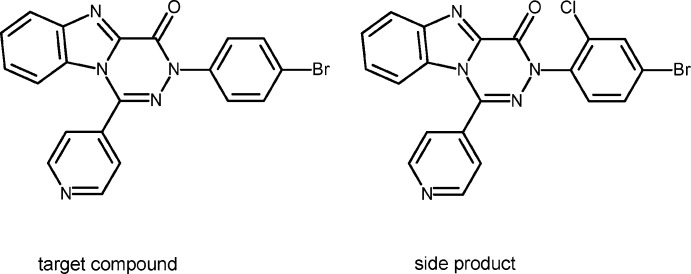


## Supra­molecular features   

The packing of (**1**) and (**2**) is dominated by π–π inter­actions. In (**1**), the 13-membered rings related by a centre of inversion are stacked with a distance of 3.513 (2) Å between the centroids of two five-membered rings (symmetry operator: 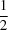 − *x*, 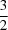 − *y*, 1 − *z*). In (**2**), the six-membered ring C14*A–*C19*A* shows a short π–π inter­action of 3.848 (3) Å with its inversion-related equivalent (symmetry operator: 2 − *x*, 1 − *y*, 1 − *z*). In addition, weak C—H⋯O and C—H⋯Br hydrogen bonds stabilize the crystal packing in (**1**) (see Table 2 ▸).

## Database survey   

Two similar structures have been published previously (Abu Thaher *et al.*, 2016*a* ▸,*b* ▸). All crystal structures show the typical π–π inter­action of the fused 13-membered ring system. The angles between the least-squares planes through the pyridine ring and the 13-membered ring vary from 50.38 (17) to 79.98 (7)°, probably depending on the mol­ecular packing, while the angles between the substituted phenyl ring and the 13-membered ring range from 43.13 (15) to 78.64 (9)° depending on the size of the substituent.

## Synthesis and crystallization   

50.4 mg of NaH was added slowly to a solution of 399.4 mg of ethyl-2-benzimidazolcarboxyl­ate in 30 ml dry THF and stirring continued at 298 K for 20 min. Then, 694 mg of *N*-(4-bromo­phen­yl)-4-pyridine­carbohydrazonoyl chloride·HCl was added slowly in a portion-wise manner; in parallel 0.5 ml of Et_3_N was added dropwise. The reaction was stirred overnight (about 12 h); the reaction mixture was filtered and concentrated under vacuum. The solid residue was purified by column chromatography (SiO_2_, hepta­ne:ethyl acetate; 2:1, then 1:1). Suitable crystals for X-ray were obtained by slow evaporation of hepta­ne/ethyl acetate (1:1).

## Refinement   

Crystal data, data collection and structure refinement details are summarized in Table 3 ▸. Hydrogen atoms attached to carbons were placed at calculated positions with C—H = 0.95 Å (aromatic) or 0.98–0.99 Å (C*sp* ^3^ atom). All H atoms were refined in the riding-model approximation with isotropic displacement parameters (set at 1.2–1.5 times of the *U*_eq_ of the parent atom). The s.o.f. for the chlorine atom in (**1**) was initially refined and then fixed at 0.125.

## Supplementary Material

Crystal structure: contains datablock(s) 1, 2, global. DOI: 10.1107/S2056989017011732/bt6995sup1.cif

Structure factors: contains datablock(s) 1. DOI: 10.1107/S2056989017011732/bt69951sup2.hkl

Structure factors: contains datablock(s) 2. DOI: 10.1107/S2056989017011732/bt69952sup3.hkl

CCDC references: 1568130, 1568129

Additional supporting information:  crystallographic information; 3D view; checkCIF report

## Figures and Tables

**Table 1:** Torsion angles (°)

Compound	N13—N1—C14—C15	N13—C12—C21—C26
(**1**)	−42.3 (4)	53.6 (5)
(**2*A***)	−53.3 (5)	−45.9 (6)
(**2*B***)	−53.7 (5)	−56.8 (6)

**Table 2:** Hydrogen-bond geometry (Å, °) for (**1**)

*D*—H⋯*A*	*D*—H	H⋯*A*	*D*⋯*A*	*D*—H⋯*A*
C6—H6⋯O20^i^	0.95	2.53	3.258 (5)	134
C15—H15⋯O20^ii^	0.95	2.30	3.219 (5)	162
C23—H23⋯Br1^iii^	0.95	2.97	3.417 (4)	110

Symmetry codes: (i) 
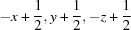
; (ii) 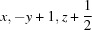; (iii) 
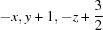
.

**Table 3:** Experimental details

	(**1**)	(**2**)
Crystal data		
Chemical formula	C_20_H_12_BrN_5_O·0.143C_20_H_11_BrClN_5_O	C_20_H_11_BrClN_5_O
*M*_r_	422.56	452.70
Crystal system, space group	Monoclinic, *C*2/*c*	Monoclinic, *P*2_1_/*c*
Temperature (K)	193	173
*a*, *b*, *c* (Å)	25.7608 (18), 11.0507 (5), 12.2709 (10)	7.1074 (7), 32.754 (3), 16.1505 (15)
β (°)	90.955 (6)	98.914 (3)
*V* (Å^3^)	3492.7 (4)	3714.4 (6)
*Z*	8	8
Radiation type	Mo *K*α	Mo *K*α
μ (mm^−1^)	2.39	2.38
Crystal size (mm)	0.28 × 0.26 × 0.12	0.28 × 0.03 × 0.02

Data collection		
Diffractometer	Stoe IPDS 2T	Bruker SMART APEXII
Absorption correction	Integration (*X-RED32*; Stoe & Cie 2006 ▸)	Multi-scan (*SADABS*; Bruker, 2000 ▸)
*T*_min_, *T*_max_	0.488, 0.744	0.872, 0.947
No. of measured, independent and observed [*I* > 2σ(*I*)] reflections	11699, 4317, 2369	31679, 8805, 3614
*R*_int_	0.041	0.152
(sin θ/λ)_max_ (Å^−1^)	0.666	0.657

Refinement		
*R*[*F* ^2^ > 2σ(*F* ^2^)], *wR*(*F* ^2^), *S*	0.051, 0.142, 1.03	0.047, 0.089, 0.72
No. of reflections	4317	8805
No. of parameters	253	505
H-atom treatment	H-atom parameters constrained	H-atom parameters constrained
Δρ_max_, Δρ_min_ (e Å^−3^)	0.65, −0.69	0.42, −0.49

Computer programs: *X-AREA* and *X-RED32* (Stoe & Cie 2006 ▸), *SMART* and *SAINT* (Bruker, 1997 ▸), *SHELXT* (Sheldrick, 2015 ▸a) and *SHELXL2014* (Sheldrick, 2015 ▸b).

## supplementary crystallographic information

### 3-(4-Bromo-2-chlorophenyl)-1-(pyridin-4-yl)benzo[4,5]imidazo[1,2-d][1,2,4]triazin-4(3H)-one–3-(4-bromophenyl)-1-(pyridin-4-yl)benzo[4,5]imidazo[1,2-d][1,2,4]triazin-4(3H)-one (1/7) (1) Crystal data

**Table:**

C_20_H_12_BrN_5_O·0.143C_20_H_11_BrClN_5_O	*F*(000) = 1696
*M**_r_* = 422.56	*D*_x_ = 1.607 Mg m^−^^3^
Monoclinic, *C*2/*c*	Mo *K*α radiation, λ = 0.71073 Å
*a* = 25.7608 (18) Å	Cell parameters from 9637 reflections
*b* = 11.0507 (5) Å	θ = 2.0–28.2°
*c* = 12.2709 (10) Å	µ = 2.39 mm^−^^1^
β = 90.955 (6)°	*T* = 193 K
*V* = 3492.7 (4) Å^3^	Needle, colourless
*Z* = 8	0.28 × 0.26 × 0.12 mm

### 3-(4-Bromo-2-chlorophenyl)-1-(pyridin-4-yl)benzo[4,5]imidazo[1,2-d][1,2,4]triazin-4(3H)-one–3-(4-bromophenyl)-1-(pyridin-4-yl)benzo[4,5]imidazo[1,2-d][1,2,4]triazin-4(3H)-one (1/7) (1) Data collection

**Table:**

Stoe IPDS 2T diffractometer	4317 independent reflections
Radiation source: sealed X-ray tube, 12 x 0.4 mm long-fine focus	2369 reflections with *I* > 2σ(*I*)
Detector resolution: 6.67 pixels mm^-1^	*R*_int_ = 0.041
rotation method scans	θ_max_ = 28.3°, θ_min_ = 2.6°
Absorption correction: integration (X-RED32; Stoe & Cie 2006)	*h* = −34→34
*T*_min_ = 0.488, *T*_max_ = 0.744	*k* = −14→14
11699 measured reflections	*l* = −16→16

### 3-(4-Bromo-2-chlorophenyl)-1-(pyridin-4-yl)benzo[4,5]imidazo[1,2-d][1,2,4]triazin-4(3H)-one–3-(4-bromophenyl)-1-(pyridin-4-yl)benzo[4,5]imidazo[1,2-d][1,2,4]triazin-4(3H)-one (1/7) (1) Refinement

**Table:**

Refinement on *F*^2^	0 restraints
Least-squares matrix: full	Hydrogen site location: inferred from neighbouring sites
*R*[*F*^2^ > 2σ(*F*^2^)] = 0.051	H-atom parameters constrained
*wR*(*F*^2^) = 0.142	*w* = 1/[σ^2^(*F*_o_^2^) + (0.0663*P*)^2^ + 0.855*P*] where *P* = (*F*_o_^2^ + 2*F*_c_^2^)/3
*S* = 1.03	(Δ/σ)_max_ < 0.001
4317 reflections	Δρ_max_ = 0.65 e Å^−^^3^
253 parameters	Δρ_min_ = −0.69 e Å^−^^3^

### 3-(4-Bromo-2-chlorophenyl)-1-(pyridin-4-yl)benzo[4,5]imidazo[1,2-d][1,2,4]triazin-4(3H)-one–3-(4-bromophenyl)-1-(pyridin-4-yl)benzo[4,5]imidazo[1,2-d][1,2,4]triazin-4(3H)-one (1/7) (1) Special details

**Table:**

Geometry. All esds (except the esd in the dihedral angle between two l.s. planes) are estimated using the full covariance matrix. The cell esds are taken into account individually in the estimation of esds in distances, angles and torsion angles; correlations between esds in cell parameters are only used when they are defined by crystal symmetry. An approximate (isotropic) treatment of cell esds is used for estimating esds involving l.s. planes.

### 3-(4-Bromo-2-chlorophenyl)-1-(pyridin-4-yl)benzo[4,5]imidazo[1,2-d][1,2,4]triazin-4(3H)-one–3-(4-bromophenyl)-1-(pyridin-4-yl)benzo[4,5]imidazo[1,2-d][1,2,4]triazin-4(3H)-one (1/7) (1) Fractional atomic coordinates and isotropic or equivalent isotropic displacement parameters (Å^2^)

**Table:**

	*x*	*y*	*z*	*U*_iso_*/*U*_eq_	Occ. (<1)
Br1	−0.02345 (2)	0.15248 (4)	0.54904 (4)	0.06608 (19)	
N1	0.11796 (12)	0.5865 (3)	0.4925 (2)	0.0379 (7)	
C2	0.14764 (14)	0.6114 (4)	0.4030 (3)	0.0390 (8)	
C3	0.17585 (14)	0.7263 (4)	0.4112 (3)	0.0399 (8)	
N4	0.20640 (12)	0.7717 (3)	0.3376 (3)	0.0467 (8)	
C5	0.22548 (14)	0.8765 (4)	0.3860 (3)	0.0448 (9)	
C6	0.25926 (16)	0.9629 (5)	0.3428 (4)	0.0548 (11)	
H6	0.2727	0.9540	0.2717	0.066*	
C7	0.27228 (16)	1.0608 (5)	0.4064 (4)	0.0580 (12)	
H7	0.2945	1.1213	0.3779	0.070*	
C8	0.25371 (17)	1.0735 (4)	0.5117 (4)	0.0558 (11)	
H8	0.2642	1.1420	0.5534	0.067*	
C9	0.22053 (15)	0.9903 (4)	0.5579 (3)	0.0483 (10)	
H9	0.2084	0.9987	0.6302	0.058*	
C10	0.20604 (14)	0.8931 (4)	0.4914 (3)	0.0421 (9)	
N11	0.17314 (11)	0.7939 (3)	0.5062 (2)	0.0366 (7)	
C12	0.14121 (14)	0.7566 (3)	0.5906 (3)	0.0361 (8)	
N13	0.11540 (11)	0.6577 (3)	0.5851 (2)	0.0374 (6)	
C14	0.08654 (14)	0.4794 (3)	0.5007 (3)	0.0385 (8)	
C15	0.08749 (17)	0.4173 (4)	0.5986 (3)	0.0481 (9)	
H15	0.1104	0.4414	0.6560	0.058*	
C16	0.05456 (17)	0.3193 (4)	0.6119 (4)	0.0523 (10)	
H16	0.0545	0.2761	0.6788	0.063*	
C17	0.02186 (16)	0.2848 (4)	0.5275 (4)	0.0485 (9)	
C18	0.02157 (15)	0.3456 (4)	0.4294 (3)	0.0467 (9)	
H18	−0.0009	0.3203	0.3717	0.056*	
C19	0.05422 (15)	0.4436 (4)	0.4156 (3)	0.0441 (9)	
H19	0.0544	0.4860	0.3483	0.053*	0.875
O20	0.15031 (10)	0.5434 (3)	0.3248 (2)	0.0490 (7)	
Cl1	0.0394 (3)	0.5181 (8)	0.3084 (6)	0.0457 (17)	0.125
C21	0.13313 (13)	0.8320 (3)	0.6879 (3)	0.0366 (8)	
C22	0.11511 (16)	0.9496 (4)	0.6782 (3)	0.0461 (9)	
H22	0.1102	0.9862	0.6088	0.055*	
C23	0.10454 (18)	1.0123 (4)	0.7731 (4)	0.0550 (11)	
H23	0.0923	1.0931	0.7663	0.066*	
N24	0.11042 (14)	0.9671 (3)	0.8731 (3)	0.0533 (9)	
C25	0.12787 (16)	0.8544 (4)	0.8800 (3)	0.0479 (9)	
H25	0.1328	0.8206	0.9506	0.057*	
C26	0.13934 (14)	0.7828 (3)	0.7911 (3)	0.0414 (8)	
H26	0.1512	0.7020	0.8006	0.050*	

### 3-(4-Bromo-2-chlorophenyl)-1-(pyridin-4-yl)benzo[4,5]imidazo[1,2-d][1,2,4]triazin-4(3H)-one–3-(4-bromophenyl)-1-(pyridin-4-yl)benzo[4,5]imidazo[1,2-d][1,2,4]triazin-4(3H)-one (1/7) (1) Atomic displacement parameters (Å^2^)

**Table:**

	*U*^11^	*U*^22^	*U*^33^	*U*^12^	*U*^13^	*U*^23^
Br1	0.0666 (3)	0.0435 (2)	0.0878 (4)	−0.0127 (2)	−0.0093 (2)	0.0036 (2)
N1	0.0417 (16)	0.0385 (16)	0.0337 (15)	0.0010 (14)	0.0011 (13)	−0.0041 (13)
C2	0.0370 (19)	0.051 (2)	0.0291 (18)	0.0038 (17)	0.0010 (14)	−0.0004 (16)
C3	0.0360 (18)	0.051 (2)	0.0334 (18)	0.0052 (17)	0.0037 (15)	0.0040 (16)
N4	0.0416 (17)	0.059 (2)	0.0402 (17)	0.0040 (16)	0.0064 (14)	0.0069 (16)
C5	0.0339 (18)	0.060 (3)	0.040 (2)	0.0046 (18)	0.0047 (15)	0.0128 (18)
C6	0.042 (2)	0.072 (3)	0.051 (2)	0.000 (2)	0.0030 (18)	0.023 (2)
C7	0.043 (2)	0.066 (3)	0.066 (3)	−0.008 (2)	−0.001 (2)	0.029 (2)
C8	0.049 (2)	0.056 (3)	0.063 (3)	−0.013 (2)	−0.006 (2)	0.015 (2)
C9	0.044 (2)	0.054 (2)	0.047 (2)	−0.0078 (19)	−0.0043 (17)	0.0080 (19)
C10	0.0340 (18)	0.046 (2)	0.047 (2)	0.0005 (16)	0.0012 (16)	0.0134 (17)
N11	0.0346 (15)	0.0443 (17)	0.0309 (14)	−0.0018 (14)	0.0033 (12)	0.0035 (13)
C12	0.0374 (18)	0.0374 (19)	0.0336 (18)	0.0012 (16)	0.0028 (14)	0.0028 (15)
N13	0.0421 (16)	0.0377 (16)	0.0325 (14)	0.0008 (15)	0.0029 (12)	−0.0019 (13)
C14	0.0402 (19)	0.0352 (18)	0.0401 (19)	0.0040 (16)	0.0012 (15)	−0.0012 (15)
C15	0.056 (2)	0.047 (2)	0.041 (2)	−0.0043 (19)	−0.0080 (18)	0.0052 (17)
C16	0.061 (3)	0.045 (2)	0.051 (2)	−0.006 (2)	−0.007 (2)	0.0074 (18)
C17	0.045 (2)	0.038 (2)	0.063 (3)	0.0010 (18)	0.0004 (19)	−0.0059 (19)
C18	0.042 (2)	0.048 (2)	0.050 (2)	−0.0033 (19)	−0.0043 (17)	−0.0087 (19)
C19	0.044 (2)	0.048 (2)	0.0399 (19)	0.0084 (18)	−0.0026 (16)	−0.0065 (17)
O20	0.0469 (15)	0.0637 (18)	0.0365 (13)	−0.0008 (14)	0.0049 (12)	−0.0113 (13)
Cl1	0.044 (4)	0.055 (5)	0.038 (4)	0.007 (3)	0.000 (3)	0.008 (3)
C21	0.0357 (17)	0.0354 (19)	0.0387 (18)	−0.0030 (15)	0.0029 (14)	−0.0012 (15)
C22	0.057 (2)	0.038 (2)	0.044 (2)	0.0027 (18)	−0.0002 (18)	−0.0005 (16)
C23	0.066 (3)	0.038 (2)	0.061 (3)	0.003 (2)	−0.002 (2)	−0.0052 (19)
N24	0.055 (2)	0.050 (2)	0.054 (2)	−0.0016 (17)	0.0052 (17)	−0.0159 (17)
C25	0.051 (2)	0.049 (2)	0.043 (2)	−0.006 (2)	0.0061 (17)	−0.0021 (19)
C26	0.045 (2)	0.040 (2)	0.0388 (19)	0.0022 (17)	0.0018 (16)	−0.0001 (16)

### 3-(4-Bromo-2-chlorophenyl)-1-(pyridin-4-yl)benzo[4,5]imidazo[1,2-d][1,2,4]triazin-4(3H)-one–3-(4-bromophenyl)-1-(pyridin-4-yl)benzo[4,5]imidazo[1,2-d][1,2,4]triazin-4(3H)-one (1/7) (1) Geometric parameters (Å, º)

**Table:**

Br1—C17	1.892 (4)	C14—C19	1.381 (5)
N1—C2	1.377 (4)	C14—C15	1.384 (5)
N1—N13	1.384 (4)	C15—C16	1.387 (6)
N1—C14	1.439 (5)	C15—H15	0.9500
C2—O20	1.221 (4)	C16—C17	1.378 (6)
C2—C3	1.465 (5)	C16—H16	0.9500
C3—N4	1.308 (4)	C17—C18	1.377 (6)
C3—N11	1.388 (5)	C18—C19	1.384 (6)
N4—C5	1.387 (5)	C18—H18	0.9500
C5—C6	1.401 (6)	C19—Cl1	1.593 (8)
C5—C10	1.407 (5)	C19—H19	0.9500
C6—C7	1.372 (7)	Cl1—Cl1^i^	2.467 (15)
C6—H6	0.9500	C21—C22	1.384 (5)
C7—C8	1.392 (7)	C21—C26	1.385 (5)
C7—H7	0.9500	C22—C23	1.386 (6)
C8—C9	1.384 (6)	C22—H22	0.9500
C8—H8	0.9500	C23—N24	1.331 (6)
C9—C10	1.396 (6)	C23—H23	0.9500
C9—H9	0.9500	N24—C25	1.327 (5)
C10—N11	1.399 (5)	C25—C26	1.383 (5)
N11—C12	1.395 (4)	C25—H25	0.9500
C12—N13	1.281 (5)	C26—H26	0.9500
C12—C21	1.474 (5)		
			
C2—N1—N13	125.2 (3)	C19—C14—N1	121.0 (3)
C2—N1—C14	122.7 (3)	C15—C14—N1	117.8 (3)
N13—N1—C14	112.1 (3)	C14—C15—C16	119.2 (4)
O20—C2—N1	122.9 (4)	C14—C15—H15	120.4
O20—C2—C3	123.6 (3)	C16—C15—H15	120.4
N1—C2—C3	113.6 (3)	C17—C16—C15	119.7 (4)
N4—C3—N11	114.4 (4)	C17—C16—H16	120.2
N4—C3—C2	126.1 (3)	C15—C16—H16	120.2
N11—C3—C2	119.5 (3)	C18—C17—C16	121.1 (4)
C3—N4—C5	103.8 (3)	C18—C17—Br1	120.3 (3)
N4—C5—C6	128.7 (4)	C16—C17—Br1	118.6 (3)
N4—C5—C10	111.9 (3)	C17—C18—C19	119.6 (4)
C6—C5—C10	119.4 (4)	C17—C18—H18	120.2
C7—C6—C5	118.0 (4)	C19—C18—H18	120.2
C7—C6—H6	121.0	C14—C19—C18	119.4 (4)
C5—C6—H6	121.0	C14—C19—Cl1	127.5 (4)
C6—C7—C8	121.5 (4)	C18—C19—Cl1	111.6 (4)
C6—C7—H7	119.3	C14—C19—H19	120.3
C8—C7—H7	119.3	C18—C19—H19	120.3
C9—C8—C7	122.5 (5)	C19—Cl1—Cl1^i^	131.6 (5)
C9—C8—H8	118.7	C22—C21—C26	118.7 (3)
C7—C8—H8	118.7	C22—C21—C12	120.9 (3)
C8—C9—C10	115.6 (4)	C26—C21—C12	120.2 (3)
C8—C9—H9	122.2	C21—C22—C23	117.9 (4)
C10—C9—H9	122.2	C21—C22—H22	121.1
C9—C10—N11	133.1 (3)	C23—C22—H22	121.1
C9—C10—C5	122.8 (4)	N24—C23—C22	124.5 (4)
N11—C10—C5	104.1 (3)	N24—C23—H23	117.8
C3—N11—C12	120.2 (3)	C22—C23—H23	117.8
C3—N11—C10	105.9 (3)	C25—N24—C23	116.4 (3)
C12—N11—C10	133.9 (3)	N24—C25—C26	124.3 (4)
N13—C12—N11	121.7 (3)	N24—C25—H25	117.9
N13—C12—C21	116.4 (3)	C26—C25—H25	117.9
N11—C12—C21	121.8 (3)	C25—C26—C21	118.3 (4)
C12—N13—N1	119.7 (3)	C25—C26—H26	120.9
C19—C14—C15	121.0 (4)	C21—C26—H26	120.9
			
N13—N1—C2—O20	176.9 (3)	N11—C12—N13—N1	−1.5 (5)
C14—N1—C2—O20	−1.5 (6)	C21—C12—N13—N1	175.2 (3)
N13—N1—C2—C3	−2.5 (5)	C2—N1—N13—C12	1.5 (5)
C14—N1—C2—C3	179.0 (3)	C14—N1—N13—C12	−179.9 (3)
O20—C2—C3—N4	1.0 (6)	C2—N1—C14—C19	−47.3 (5)
N1—C2—C3—N4	−179.5 (4)	N13—N1—C14—C19	134.1 (3)
O20—C2—C3—N11	−175.8 (4)	C2—N1—C14—C15	136.3 (4)
N1—C2—C3—N11	3.7 (5)	N13—N1—C14—C15	−42.3 (4)
N11—C3—N4—C5	0.0 (4)	C19—C14—C15—C16	−1.4 (6)
C2—C3—N4—C5	−176.9 (4)	N1—C14—C15—C16	174.9 (4)
C3—N4—C5—C6	−178.4 (4)	C14—C15—C16—C17	0.5 (6)
C3—N4—C5—C10	0.5 (4)	C15—C16—C17—C18	0.5 (6)
N4—C5—C6—C7	179.2 (4)	C15—C16—C17—Br1	−178.9 (3)
C10—C5—C6—C7	0.4 (6)	C16—C17—C18—C19	−0.7 (6)
C5—C6—C7—C8	1.5 (7)	Br1—C17—C18—C19	178.8 (3)
C6—C7—C8—C9	−1.2 (7)	C15—C14—C19—C18	1.3 (5)
C7—C8—C9—C10	−1.0 (6)	N1—C14—C19—C18	−175.0 (3)
C8—C9—C10—N11	−178.3 (4)	C15—C14—C19—Cl1	166.3 (5)
C8—C9—C10—C5	2.9 (6)	N1—C14—C19—Cl1	−10.0 (6)
N4—C5—C10—C9	178.3 (4)	C17—C18—C19—C14	−0.2 (6)
C6—C5—C10—C9	−2.7 (6)	C17—C18—C19—Cl1	−167.5 (4)
N4—C5—C10—N11	−0.8 (4)	C14—C19—Cl1—Cl1^i^	−150.2 (4)
C6—C5—C10—N11	178.3 (3)	C18—C19—Cl1—Cl1^i^	15.8 (7)
N4—C3—N11—C12	178.9 (3)	N13—C12—C21—C22	−120.9 (4)
C2—C3—N11—C12	−4.0 (5)	N11—C12—C21—C22	55.7 (5)
N4—C3—N11—C10	−0.5 (4)	N13—C12—C21—C26	53.6 (5)
C2—C3—N11—C10	176.7 (3)	N11—C12—C21—C26	−129.7 (4)
C9—C10—N11—C3	−178.2 (4)	C26—C21—C22—C23	0.4 (6)
C5—C10—N11—C3	0.7 (4)	C12—C21—C22—C23	175.1 (4)
C9—C10—N11—C12	2.6 (7)	C21—C22—C23—N24	−0.2 (7)
C5—C10—N11—C12	−178.5 (4)	C22—C23—N24—C25	0.3 (7)
C3—N11—C12—N13	2.9 (5)	C23—N24—C25—C26	−0.8 (6)
C10—N11—C12—N13	−178.0 (4)	N24—C25—C26—C21	1.1 (6)
C3—N11—C12—C21	−173.6 (3)	C22—C21—C26—C25	−0.9 (5)
C10—N11—C12—C21	5.5 (6)	C12—C21—C26—C25	−175.6 (3)

Symmetry code: (i) −*x*, *y*, −*z*+1/2.

### 3-(4-Bromo-2-chlorophenyl)-1-(pyridin-4-yl)benzo[4,5]imidazo[1,2-d][1,2,4]triazin-4(3H)-one–3-(4-bromophenyl)-1-(pyridin-4-yl)benzo[4,5]imidazo[1,2-d][1,2,4]triazin-4(3H)-one (1/7) (1) Hydrogen-bond geometry (Å, º)

**Table:**

*D*—H···*A*	*D*—H	H···*A*	*D*···*A*	*D*—H···*A*
C6—H6···O20^ii^	0.95	2.53	3.258 (5)	134
C15—H15···O20^iii^	0.95	2.30	3.219 (5)	162
C23—H23···Br1^iv^	0.95	2.97	3.417 (4)	110

Symmetry codes: (ii) −*x*+1/2, *y*+1/2, −*z*+1/2; (iii) *x*, −*y*+1, *z*+1/2; (iv) −*x*, *y*+1, −*z*+3/2.

### 3-(4-Bromo-2-chlorophenyl)-1-(pyridin-4-yl)benzo[4,5]imidazo[1,2-d][1,2,4]triazin-4(3H)-one (2) Crystal data

**Table:**

C_20_H_11_BrClN_5_O	*F*(000) = 1808
*M**_r_* = 452.70	*D*_x_ = 1.619 Mg m^−^^3^
Monoclinic, *P*2_1_/*c*	Mo *K*α radiation, λ = 0.71073 Å
*a* = 7.1074 (7) Å	Cell parameters from 1476 reflections
*b* = 32.754 (3) Å	θ = 2.5–19.7°
*c* = 16.1505 (15) Å	µ = 2.38 mm^−^^1^
β = 98.914 (3)°	*T* = 173 K
*V* = 3714.4 (6) Å^3^	Column, colourless
*Z* = 8	0.28 × 0.03 × 0.02 mm

### 3-(4-Bromo-2-chlorophenyl)-1-(pyridin-4-yl)benzo[4,5]imidazo[1,2-d][1,2,4]triazin-4(3H)-one (2) Data collection

**Table:**

Bruker SMART APEXII diffractometer	3614 reflections with *I* > 2σ(*I*)
Radiation source: sealed tube	*R*_int_ = 0.152
CCD scan	θ_max_ = 27.8°, θ_min_ = 1.2°
Absorption correction: multi-scan (SADABS; Bruker, 2000)	*h* = −9→9
*T*_min_ = 0.872, *T*_max_ = 0.947	*k* = −41→42
31679 measured reflections	*l* = −21→21
8805 independent reflections	

### 3-(4-Bromo-2-chlorophenyl)-1-(pyridin-4-yl)benzo[4,5]imidazo[1,2-d][1,2,4]triazin-4(3H)-one (2) Refinement

**Table:**

Refinement on *F*^2^	0 restraints
Least-squares matrix: full	Hydrogen site location: inferred from neighbouring sites
*R*[*F*^2^ > 2σ(*F*^2^)] = 0.047	H-atom parameters constrained
*wR*(*F*^2^) = 0.089	*w* = 1/[σ^2^(*F*_o_^2^) + (0.012*P*)^2^] where *P* = (*F*_o_^2^ + 2*F*_c_^2^)/3
*S* = 0.72	(Δ/σ)_max_ = 0.001
8805 reflections	Δρ_max_ = 0.42 e Å^−^^3^
505 parameters	Δρ_min_ = −0.49 e Å^−^^3^

### 3-(4-Bromo-2-chlorophenyl)-1-(pyridin-4-yl)benzo[4,5]imidazo[1,2-d][1,2,4]triazin-4(3H)-one (2) Special details

**Table:**

Geometry. All esds (except the esd in the dihedral angle between two l.s. planes) are estimated using the full covariance matrix. The cell esds are taken into account individually in the estimation of esds in distances, angles and torsion angles; correlations between esds in cell parameters are only used when they are defined by crystal symmetry. An approximate (isotropic) treatment of cell esds is used for estimating esds involving l.s. planes.

### 3-(4-Bromo-2-chlorophenyl)-1-(pyridin-4-yl)benzo[4,5]imidazo[1,2-d][1,2,4]triazin-4(3H)-one (2) Fractional atomic coordinates and isotropic or equivalent isotropic displacement parameters (Å^2^)

**Table:**

	*x*	*y*	*z*	*U*_iso_*/*U*_eq_	
Br1A	0.96493 (8)	0.44476 (2)	0.31444 (3)	0.04310 (17)	
Cl1A	1.36103 (18)	0.46317 (5)	0.63345 (8)	0.0432 (4)	
N1A	0.9964 (5)	0.43502 (10)	0.6924 (2)	0.0173 (9)	
C2A	1.0976 (6)	0.40286 (13)	0.7334 (3)	0.0176 (11)	
C3A	1.0808 (6)	0.40069 (13)	0.8227 (3)	0.0165 (11)	
N4A	1.1804 (5)	0.37751 (11)	0.8789 (2)	0.0208 (9)	
C5A	1.1249 (6)	0.38977 (13)	0.9538 (3)	0.0170 (11)	
C6A	1.1926 (6)	0.37608 (14)	1.0348 (3)	0.0236 (12)	
H6A	1.2828	0.3545	1.0443	0.028*	
C7A	1.1243 (6)	0.39487 (15)	1.1007 (3)	0.0276 (12)	
H7A	1.1682	0.3861	1.1564	0.033*	
C8A	0.9922 (6)	0.42647 (14)	1.0869 (3)	0.0260 (12)	
H8A	0.9478	0.4387	1.1336	0.031*	
C9A	0.9232 (6)	0.44073 (14)	1.0073 (3)	0.0244 (12)	
H9A	0.8356	0.4628	0.9984	0.029*	
C10A	0.9887 (6)	0.42112 (13)	0.9411 (3)	0.0173 (11)	
N11A	0.9589 (5)	0.42773 (10)	0.8541 (2)	0.0155 (9)	
C12A	0.8362 (6)	0.45263 (13)	0.8002 (3)	0.0148 (10)	
N13A	0.8534 (5)	0.45672 (10)	0.7225 (2)	0.0180 (9)	
C14A	0.9909 (6)	0.43961 (13)	0.6034 (3)	0.0179 (11)	
C15A	0.8217 (7)	0.43142 (14)	0.5505 (3)	0.0256 (12)	
H15A	0.7111	0.4242	0.5735	0.031*	
C16A	0.8132 (7)	0.43367 (13)	0.4648 (3)	0.0236 (12)	
H16A	0.6973	0.4281	0.4287	0.028*	
C17A	0.9736 (7)	0.44400 (15)	0.4324 (3)	0.0257 (12)	
C18A	1.1435 (7)	0.45322 (14)	0.4828 (3)	0.0260 (12)	
H18A	1.2525	0.4611	0.4593	0.031*	
C19A	1.1500 (6)	0.45062 (14)	0.5696 (3)	0.0218 (11)	
O20A	1.1978 (4)	0.38045 (9)	0.69899 (18)	0.0270 (8)	
C21A	0.6743 (6)	0.47361 (13)	0.8297 (3)	0.0148 (10)	
C22A	0.5556 (6)	0.45303 (14)	0.8762 (3)	0.0210 (11)	
H22A	0.5774	0.4252	0.8909	0.025*	
C23A	0.4048 (6)	0.47409 (15)	0.9007 (3)	0.0252 (12)	
H23A	0.3270	0.4600	0.9342	0.030*	
N24A	0.3612 (5)	0.51258 (13)	0.8807 (2)	0.0304 (11)	
C25A	0.4749 (7)	0.53165 (15)	0.8346 (3)	0.0297 (13)	
H25A	0.4465	0.5592	0.8189	0.036*	
C26A	0.6319 (6)	0.51357 (14)	0.8083 (3)	0.0241 (12)	
H26A	0.7091	0.5286	0.7760	0.029*	
Br1B	0.47931 (8)	0.36336 (2)	0.29334 (3)	0.03770 (16)	
Cl1B	0.84923 (18)	0.30274 (4)	0.59237 (8)	0.0411 (4)	
N1B	0.4871 (5)	0.32230 (11)	0.6617 (2)	0.0166 (9)	
C2B	0.5946 (6)	0.34732 (14)	0.7198 (3)	0.0196 (11)	
C3B	0.5793 (6)	0.33619 (14)	0.8071 (3)	0.0201 (11)	
N4B	0.6815 (5)	0.35077 (11)	0.8745 (2)	0.0201 (9)	
C5B	0.6216 (6)	0.32841 (14)	0.9391 (3)	0.0197 (11)	
C6B	0.6875 (7)	0.33113 (15)	1.0247 (3)	0.0277 (12)	
H6B	0.7827	0.3504	1.0461	0.033*	
C7B	0.6107 (7)	0.30507 (15)	1.0773 (3)	0.0303 (13)	
H7B	0.6537	0.3064	1.1359	0.036*	
C8B	0.4708 (7)	0.27673 (14)	1.0461 (3)	0.0263 (12)	
H8B	0.4211	0.2592	1.0842	0.032*	
C9B	0.4024 (6)	0.27336 (14)	0.9619 (3)	0.0220 (11)	
H9B	0.3088	0.2537	0.9410	0.026*	
C10B	0.4779 (6)	0.30041 (14)	0.9083 (3)	0.0200 (11)	
N11B	0.4492 (5)	0.30633 (10)	0.8217 (2)	0.0155 (8)	
C12B	0.3257 (6)	0.28919 (13)	0.7551 (3)	0.0170 (11)	
N13B	0.3423 (5)	0.29650 (11)	0.6775 (2)	0.0189 (9)	
C14B	0.4850 (6)	0.33054 (13)	0.5739 (3)	0.0160 (10)	
C15B	0.3200 (6)	0.34514 (13)	0.5259 (3)	0.0225 (12)	
H15B	0.2088	0.3488	0.5511	0.027*	
C16B	0.3145 (7)	0.35454 (14)	0.4419 (3)	0.0277 (12)	
H16B	0.2020	0.3650	0.4092	0.033*	
C17B	0.4782 (7)	0.34825 (14)	0.4070 (3)	0.0222 (11)	
C18B	0.6421 (6)	0.33246 (13)	0.4522 (3)	0.0216 (11)	
H18B	0.7513	0.3278	0.4263	0.026*	
C19B	0.6451 (6)	0.32348 (13)	0.5359 (3)	0.0185 (11)	
O20B	0.6995 (4)	0.37385 (9)	0.70109 (18)	0.0276 (8)	
C21B	0.1679 (6)	0.26314 (14)	0.7722 (3)	0.0195 (11)	
C22B	0.0357 (6)	0.27684 (15)	0.8214 (3)	0.0271 (12)	
H22B	0.0425	0.3036	0.8443	0.033*	
C23B	−0.1051 (7)	0.24998 (16)	0.8354 (3)	0.0347 (14)	
H23B	−0.1953	0.2596	0.8685	0.042*	
N24B	−0.1270 (6)	0.21199 (13)	0.8073 (3)	0.0349 (11)	
C25B	−0.0011 (7)	0.19985 (15)	0.7593 (3)	0.0365 (14)	
H25B	−0.0126	0.1730	0.7369	0.044*	
C26B	0.1469 (6)	0.22436 (14)	0.7400 (3)	0.0271 (12)	
H26B	0.2322	0.2143	0.7050	0.033*	

### 3-(4-Bromo-2-chlorophenyl)-1-(pyridin-4-yl)benzo[4,5]imidazo[1,2-d][1,2,4]triazin-4(3H)-one (2) Atomic displacement parameters (Å^2^)

**Table:**

	*U*^11^	*U*^22^	*U*^33^	*U*^12^	*U*^13^	*U*^23^
Br1A	0.0516 (4)	0.0607 (4)	0.0186 (3)	−0.0114 (3)	0.0105 (3)	0.0025 (3)
Cl1A	0.0284 (8)	0.0656 (11)	0.0359 (8)	−0.0056 (7)	0.0059 (7)	0.0006 (7)
N1A	0.018 (2)	0.020 (2)	0.016 (2)	0.0015 (18)	0.0088 (17)	0.0006 (16)
C2A	0.018 (3)	0.015 (3)	0.021 (3)	0.001 (2)	0.003 (2)	0.001 (2)
C3A	0.016 (2)	0.018 (3)	0.016 (3)	−0.002 (2)	0.003 (2)	−0.004 (2)
N4A	0.020 (2)	0.026 (3)	0.017 (2)	0.0050 (19)	0.0047 (18)	−0.0014 (17)
C5A	0.014 (2)	0.019 (3)	0.017 (3)	0.002 (2)	0.001 (2)	0.002 (2)
C6A	0.016 (3)	0.027 (3)	0.027 (3)	0.006 (2)	0.003 (2)	0.003 (2)
C7A	0.024 (3)	0.041 (4)	0.017 (3)	0.006 (3)	−0.002 (2)	0.006 (2)
C8A	0.024 (3)	0.034 (3)	0.021 (3)	0.002 (2)	0.008 (2)	−0.005 (2)
C9A	0.017 (3)	0.033 (3)	0.023 (3)	0.003 (2)	0.003 (2)	0.001 (2)
C10A	0.019 (3)	0.020 (3)	0.013 (2)	−0.002 (2)	0.004 (2)	0.001 (2)
N11A	0.013 (2)	0.019 (2)	0.016 (2)	0.0031 (17)	0.0055 (17)	−0.0021 (16)
C12A	0.009 (2)	0.014 (3)	0.021 (3)	0.002 (2)	0.002 (2)	−0.002 (2)
N13A	0.017 (2)	0.018 (2)	0.020 (2)	0.0024 (18)	0.0047 (18)	0.0010 (17)
C14A	0.018 (3)	0.016 (3)	0.020 (3)	0.002 (2)	0.006 (2)	0.000 (2)
C15A	0.023 (3)	0.032 (3)	0.024 (3)	−0.006 (2)	0.010 (2)	0.003 (2)
C16A	0.024 (3)	0.023 (3)	0.021 (3)	−0.004 (2)	−0.006 (2)	0.000 (2)
C17A	0.033 (3)	0.031 (3)	0.015 (2)	−0.002 (3)	0.012 (2)	0.001 (2)
C18A	0.026 (3)	0.029 (3)	0.027 (3)	−0.001 (2)	0.016 (2)	0.004 (2)
C19A	0.014 (3)	0.027 (3)	0.024 (3)	−0.003 (2)	0.000 (2)	−0.006 (2)
O20A	0.031 (2)	0.029 (2)	0.0237 (19)	0.0132 (16)	0.0131 (16)	−0.0014 (15)
C21A	0.013 (2)	0.017 (3)	0.013 (2)	0.002 (2)	−0.004 (2)	−0.0012 (19)
C22A	0.018 (3)	0.021 (3)	0.025 (3)	0.000 (2)	0.008 (2)	0.000 (2)
C23A	0.016 (3)	0.028 (3)	0.033 (3)	0.001 (2)	0.010 (2)	0.002 (2)
N24A	0.024 (2)	0.031 (3)	0.040 (3)	0.008 (2)	0.015 (2)	−0.001 (2)
C25A	0.033 (3)	0.017 (3)	0.041 (3)	0.004 (2)	0.012 (3)	0.003 (2)
C26A	0.016 (3)	0.028 (3)	0.030 (3)	0.000 (2)	0.006 (2)	0.000 (2)
Br1B	0.0445 (4)	0.0483 (4)	0.0207 (3)	0.0002 (3)	0.0061 (3)	0.0076 (3)
Cl1B	0.0281 (8)	0.0597 (11)	0.0365 (8)	0.0102 (7)	0.0084 (6)	0.0071 (7)
N1B	0.017 (2)	0.017 (2)	0.017 (2)	−0.0045 (18)	0.0068 (18)	0.0012 (17)
C2B	0.014 (3)	0.026 (3)	0.019 (3)	0.003 (2)	0.005 (2)	−0.002 (2)
C3B	0.020 (3)	0.020 (3)	0.022 (3)	−0.005 (2)	0.010 (2)	−0.002 (2)
N4B	0.021 (2)	0.021 (2)	0.018 (2)	−0.0038 (18)	0.0020 (18)	0.0006 (17)
C5B	0.018 (3)	0.022 (3)	0.018 (3)	0.005 (2)	−0.001 (2)	0.001 (2)
C6B	0.023 (3)	0.034 (3)	0.026 (3)	−0.001 (2)	0.001 (2)	−0.007 (2)
C7B	0.034 (3)	0.042 (4)	0.012 (3)	0.007 (3)	−0.004 (2)	0.003 (2)
C8B	0.031 (3)	0.026 (3)	0.026 (3)	0.005 (3)	0.018 (2)	0.008 (2)
C9B	0.028 (3)	0.020 (3)	0.020 (3)	−0.001 (2)	0.009 (2)	0.002 (2)
C10B	0.022 (3)	0.022 (3)	0.017 (3)	0.005 (2)	0.004 (2)	0.003 (2)
N11B	0.015 (2)	0.019 (2)	0.013 (2)	−0.0006 (18)	0.0045 (17)	−0.0005 (17)
C12B	0.011 (2)	0.021 (3)	0.019 (3)	0.001 (2)	0.003 (2)	0.000 (2)
N13B	0.017 (2)	0.020 (2)	0.021 (2)	−0.0023 (18)	0.0067 (18)	−0.0003 (17)
C14B	0.020 (3)	0.015 (3)	0.015 (2)	0.001 (2)	0.006 (2)	−0.0019 (19)
C15B	0.013 (3)	0.031 (3)	0.025 (3)	0.005 (2)	0.008 (2)	−0.001 (2)
C16B	0.022 (3)	0.036 (3)	0.025 (3)	0.007 (2)	0.002 (2)	0.002 (2)
C17B	0.031 (3)	0.021 (3)	0.015 (3)	−0.002 (2)	0.008 (2)	0.002 (2)
C18B	0.018 (3)	0.028 (3)	0.019 (3)	0.000 (2)	0.007 (2)	0.000 (2)
C19B	0.018 (3)	0.018 (3)	0.020 (3)	0.004 (2)	0.004 (2)	0.003 (2)
O20B	0.030 (2)	0.030 (2)	0.0238 (19)	−0.0150 (17)	0.0078 (16)	0.0011 (15)
C21B	0.021 (3)	0.017 (3)	0.020 (3)	−0.005 (2)	0.003 (2)	0.001 (2)
C22B	0.026 (3)	0.027 (3)	0.030 (3)	0.001 (2)	0.011 (2)	−0.003 (2)
C23B	0.021 (3)	0.042 (4)	0.044 (4)	0.002 (3)	0.015 (3)	0.003 (3)
N24B	0.027 (3)	0.037 (3)	0.043 (3)	−0.007 (2)	0.013 (2)	0.000 (2)
C25B	0.045 (4)	0.023 (3)	0.043 (4)	−0.008 (3)	0.015 (3)	−0.011 (3)
C26B	0.026 (3)	0.029 (3)	0.029 (3)	−0.005 (3)	0.013 (2)	−0.001 (2)

### 3-(4-Bromo-2-chlorophenyl)-1-(pyridin-4-yl)benzo[4,5]imidazo[1,2-d][1,2,4]triazin-4(3H)-one (2) Geometric parameters (Å, º)

**Table:**

Br1A—C17A	1.896 (4)	Br1B—C17B	1.902 (4)
Cl1A—C19A	1.733 (4)	Cl1B—C19B	1.729 (4)
N1A—C2A	1.385 (5)	N1B—C2B	1.383 (5)
N1A—N13A	1.389 (4)	N1B—N13B	1.385 (4)
N1A—C14A	1.439 (5)	N1B—C14B	1.442 (5)
C2A—O20A	1.215 (5)	C2B—O20B	1.213 (5)
C2A—C3A	1.467 (6)	C2B—C3B	1.476 (6)
C3A—N4A	1.305 (5)	C3B—N4B	1.302 (5)
C3A—N11A	1.389 (5)	C3B—N11B	1.391 (5)
N4A—C5A	1.389 (5)	N4B—C5B	1.394 (5)
C5A—C6A	1.396 (6)	C5B—C6B	1.391 (6)
C5A—C10A	1.404 (6)	C5B—C10B	1.405 (6)
C6A—C7A	1.381 (6)	C6B—C7B	1.375 (6)
C6A—H6A	0.9500	C6B—H6B	0.9500
C7A—C8A	1.392 (6)	C7B—C8B	1.396 (6)
C7A—H7A	0.9500	C7B—H7B	0.9500
C8A—C9A	1.384 (6)	C8B—C9B	1.376 (6)
C8A—H8A	0.9500	C8B—H8B	0.9500
C9A—C10A	1.387 (6)	C9B—C10B	1.402 (6)
C9A—H9A	0.9500	C9B—H9B	0.9500
C10A—N11A	1.405 (5)	C10B—N11B	1.395 (5)
N11A—C12A	1.396 (5)	N11B—C12B	1.397 (5)
C12A—N13A	1.285 (5)	C12B—N13B	1.299 (5)
C12A—C21A	1.482 (5)	C12B—C21B	1.469 (6)
C14A—C19A	1.378 (6)	C14B—C15B	1.386 (6)
C14A—C15A	1.390 (6)	C14B—C19B	1.393 (5)
C15A—C16A	1.377 (6)	C15B—C16B	1.387 (6)
C15A—H15A	0.9500	C15B—H15B	0.9500
C16A—C17A	1.369 (6)	C16B—C17B	1.385 (6)
C16A—H16A	0.9500	C16B—H16B	0.9500
C17A—C18A	1.382 (6)	C17B—C18B	1.376 (6)
C18A—C19A	1.397 (6)	C18B—C19B	1.380 (5)
C18A—H18A	0.9500	C18B—H18B	0.9500
C21A—C26A	1.375 (6)	C21B—C26B	1.372 (6)
C21A—C22A	1.388 (5)	C21B—C22B	1.395 (6)
C22A—C23A	1.383 (6)	C22B—C23B	1.377 (6)
C22A—H22A	0.9500	C22B—H22B	0.9500
C23A—N24A	1.326 (5)	C23B—N24B	1.326 (6)
C23A—H23A	0.9500	C23B—H23B	0.9500
N24A—C25A	1.336 (5)	N24B—C25B	1.332 (6)
C25A—C26A	1.387 (6)	C25B—C26B	1.396 (6)
C25A—H25A	0.9500	C25B—H25B	0.9500
C26A—H26A	0.9500	C26B—H26B	0.9500
			
C2A—N1A—N13A	125.1 (3)	C2B—N1B—N13B	125.8 (3)
C2A—N1A—C14A	119.2 (3)	C2B—N1B—C14B	118.5 (4)
N13A—N1A—C14A	112.9 (3)	N13B—N1B—C14B	113.6 (3)
O20A—C2A—N1A	122.6 (4)	O20B—C2B—N1B	123.5 (4)
O20A—C2A—C3A	124.2 (4)	O20B—C2B—C3B	123.6 (4)
N1A—C2A—C3A	113.0 (4)	N1B—C2B—C3B	112.8 (4)
N4A—C3A—N11A	114.8 (4)	N4B—C3B—N11B	114.5 (4)
N4A—C3A—C2A	126.5 (4)	N4B—C3B—C2B	126.4 (4)
N11A—C3A—C2A	118.6 (4)	N11B—C3B—C2B	119.0 (4)
C3A—N4A—C5A	104.0 (4)	C3B—N4B—C5B	103.7 (4)
N4A—C5A—C6A	128.3 (4)	C6B—C5B—N4B	127.9 (4)
N4A—C5A—C10A	111.5 (4)	C6B—C5B—C10B	120.5 (4)
C6A—C5A—C10A	120.1 (4)	N4B—C5B—C10B	111.6 (4)
C7A—C6A—C5A	117.9 (4)	C7B—C6B—C5B	117.9 (4)
C7A—C6A—H6A	121.1	C7B—C6B—H6B	121.0
C5A—C6A—H6A	121.1	C5B—C6B—H6B	121.0
C6A—C7A—C8A	121.1 (4)	C6B—C7B—C8B	121.2 (4)
C6A—C7A—H7A	119.5	C6B—C7B—H7B	119.4
C8A—C7A—H7A	119.5	C8B—C7B—H7B	119.4
C9A—C8A—C7A	122.2 (4)	C9B—C8B—C7B	122.2 (4)
C9A—C8A—H8A	118.9	C9B—C8B—H8B	118.9
C7A—C8A—H8A	118.9	C7B—C8B—H8B	118.9
C8A—C9A—C10A	116.5 (4)	C8B—C9B—C10B	116.7 (4)
C8A—C9A—H9A	121.7	C8B—C9B—H9B	121.7
C10A—C9A—H9A	121.7	C10B—C9B—H9B	121.7
C9A—C10A—C5A	122.2 (4)	N11B—C10B—C9B	134.1 (4)
C9A—C10A—N11A	133.0 (4)	N11B—C10B—C5B	104.5 (4)
C5A—C10A—N11A	104.6 (4)	C9B—C10B—C5B	121.4 (4)
C3A—N11A—C12A	120.6 (4)	C3B—N11B—C10B	105.7 (4)
C3A—N11A—C10A	105.1 (3)	C3B—N11B—C12B	120.5 (4)
C12A—N11A—C10A	134.2 (4)	C10B—N11B—C12B	133.9 (4)
N13A—C12A—N11A	121.6 (4)	N13B—C12B—N11B	121.9 (4)
N13A—C12A—C21A	117.5 (4)	N13B—C12B—C21B	118.3 (4)
N11A—C12A—C21A	120.8 (4)	N11B—C12B—C21B	119.7 (4)
C12A—N13A—N1A	118.3 (3)	C12B—N13B—N1B	118.1 (4)
C19A—C14A—C15A	119.4 (4)	C15B—C14B—C19B	119.3 (4)
C19A—C14A—N1A	121.7 (4)	C15B—C14B—N1B	119.5 (4)
C15A—C14A—N1A	118.8 (4)	C19B—C14B—N1B	121.1 (4)
C16A—C15A—C14A	120.5 (4)	C14B—C15B—C16B	121.1 (4)
C16A—C15A—H15A	119.8	C14B—C15B—H15B	119.4
C14A—C15A—H15A	119.8	C16B—C15B—H15B	119.4
C17A—C16A—C15A	119.2 (4)	C17B—C16B—C15B	117.9 (4)
C17A—C16A—H16A	120.4	C17B—C16B—H16B	121.1
C15A—C16A—H16A	120.4	C15B—C16B—H16B	121.1
C16A—C17A—C18A	122.1 (4)	C18B—C17B—C16B	122.4 (4)
C16A—C17A—Br1A	119.3 (4)	C18B—C17B—Br1B	118.7 (3)
C18A—C17A—Br1A	118.6 (3)	C16B—C17B—Br1B	118.9 (4)
C17A—C18A—C19A	117.9 (4)	C17B—C18B—C19B	118.9 (4)
C17A—C18A—H18A	121.0	C17B—C18B—H18B	120.5
C19A—C18A—H18A	121.0	C19B—C18B—H18B	120.5
C14A—C19A—C18A	120.8 (4)	C18B—C19B—C14B	120.4 (4)
C14A—C19A—Cl1A	120.9 (3)	C18B—C19B—Cl1B	119.0 (3)
C18A—C19A—Cl1A	118.3 (3)	C14B—C19B—Cl1B	120.6 (3)
C26A—C21A—C22A	118.1 (4)	C26B—C21B—C22B	118.0 (4)
C26A—C21A—C12A	120.7 (4)	C26B—C21B—C12B	120.5 (4)
C22A—C21A—C12A	121.1 (4)	C22B—C21B—C12B	121.4 (4)
C23A—C22A—C21A	118.4 (4)	C23B—C22B—C21B	117.3 (5)
C23A—C22A—H22A	120.8	C23B—C22B—H22B	121.3
C21A—C22A—H22A	120.8	C21B—C22B—H22B	121.3
N24A—C23A—C22A	124.6 (4)	N24B—C23B—C22B	126.3 (5)
N24A—C23A—H23A	117.7	N24B—C23B—H23B	116.8
C22A—C23A—H23A	117.7	C22B—C23B—H23B	116.8
C23A—N24A—C25A	116.2 (4)	C23B—N24B—C25B	115.2 (4)
N24A—C25A—C26A	123.8 (5)	N24B—C25B—C26B	123.7 (5)
N24A—C25A—H25A	118.1	N24B—C25B—H25B	118.1
C26A—C25A—H25A	118.1	C26B—C25B—H25B	118.1
C21A—C26A—C25A	119.0 (4)	C21B—C26B—C25B	119.3 (4)
C21A—C26A—H26A	120.5	C21B—C26B—H26B	120.3
C25A—C26A—H26A	120.5	C25B—C26B—H26B	120.3
			
N13A—N1A—C2A—O20A	167.2 (4)	N13B—N1B—C2B—O20B	−168.4 (4)
C14A—N1A—C2A—O20A	7.3 (6)	C14B—N1B—C2B—O20B	−6.1 (6)
N13A—N1A—C2A—C3A	−16.7 (6)	N13B—N1B—C2B—C3B	16.1 (6)
C14A—N1A—C2A—C3A	−176.5 (4)	C14B—N1B—C2B—C3B	178.3 (4)
O20A—C2A—C3A—N4A	6.3 (8)	O20B—C2B—C3B—N4B	−4.7 (8)
N1A—C2A—C3A—N4A	−169.7 (4)	N1B—C2B—C3B—N4B	170.8 (4)
O20A—C2A—C3A—N11A	−179.0 (4)	O20B—C2B—C3B—N11B	177.6 (4)
N1A—C2A—C3A—N11A	4.9 (6)	N1B—C2B—C3B—N11B	−6.8 (6)
N11A—C3A—N4A—C5A	−0.7 (5)	N11B—C3B—N4B—C5B	2.0 (5)
C2A—C3A—N4A—C5A	174.1 (4)	C2B—C3B—N4B—C5B	−175.7 (4)
C3A—N4A—C5A—C6A	−177.2 (5)	C3B—N4B—C5B—C6B	178.7 (4)
C3A—N4A—C5A—C10A	−0.1 (5)	C3B—N4B—C5B—C10B	−1.0 (5)
N4A—C5A—C6A—C7A	175.7 (4)	N4B—C5B—C6B—C7B	−178.2 (4)
C10A—C5A—C6A—C7A	−1.3 (7)	C10B—C5B—C6B—C7B	1.4 (7)
C5A—C6A—C7A—C8A	0.0 (7)	C5B—C6B—C7B—C8B	−0.1 (7)
C6A—C7A—C8A—C9A	−0.2 (7)	C6B—C7B—C8B—C9B	−0.1 (7)
C7A—C8A—C9A—C10A	1.7 (7)	C7B—C8B—C9B—C10B	−1.1 (7)
C8A—C9A—C10A—C5A	−3.0 (7)	C8B—C9B—C10B—N11B	179.0 (4)
C8A—C9A—C10A—N11A	−176.8 (4)	C8B—C9B—C10B—C5B	2.4 (7)
N4A—C5A—C10A—C9A	−174.5 (4)	C6B—C5B—C10B—N11B	179.9 (4)
C6A—C5A—C10A—C9A	2.9 (7)	N4B—C5B—C10B—N11B	−0.4 (5)
N4A—C5A—C10A—N11A	0.8 (5)	C6B—C5B—C10B—C9B	−2.6 (7)
C6A—C5A—C10A—N11A	178.2 (4)	N4B—C5B—C10B—C9B	177.0 (4)
N4A—C3A—N11A—C12A	−176.3 (4)	N4B—C3B—N11B—C10B	−2.3 (5)
C2A—C3A—N11A—C12A	8.5 (6)	C2B—C3B—N11B—C10B	175.6 (4)
N4A—C3A—N11A—C10A	1.2 (5)	N4B—C3B—N11B—C12B	177.6 (4)
C2A—C3A—N11A—C10A	−174.0 (4)	C2B—C3B—N11B—C12B	−4.5 (6)
C9A—C10A—N11A—C3A	173.4 (5)	C9B—C10B—N11B—C3B	−175.4 (5)
C5A—C10A—N11A—C3A	−1.1 (4)	C5B—C10B—N11B—C3B	1.5 (4)
C9A—C10A—N11A—C12A	−9.6 (8)	C9B—C10B—N11B—C12B	4.6 (8)
C5A—C10A—N11A—C12A	175.9 (4)	C5B—C10B—N11B—C12B	−178.4 (4)
C3A—N11A—C12A—N13A	−12.1 (6)	C3B—N11B—C12B—N13B	8.6 (6)
C10A—N11A—C12A—N13A	171.3 (4)	C10B—N11B—C12B—N13B	−171.5 (4)
C3A—N11A—C12A—C21A	164.6 (4)	C3B—N11B—C12B—C21B	−169.4 (4)
C10A—N11A—C12A—C21A	−12.1 (7)	C10B—N11B—C12B—C21B	10.5 (7)
N11A—C12A—N13A—N1A	1.2 (6)	N11B—C12B—N13B—N1B	−0.4 (6)
C21A—C12A—N13A—N1A	−175.6 (4)	C21B—C12B—N13B—N1B	177.7 (4)
C2A—N1A—N13A—C12A	14.2 (6)	C2B—N1B—N13B—C12B	−13.0 (6)
C14A—N1A—N13A—C12A	175.2 (4)	C14B—N1B—N13B—C12B	−176.0 (4)
C2A—N1A—C14A—C19A	−69.0 (6)	C2B—N1B—C14B—C15B	−110.7 (5)
N13A—N1A—C14A—C19A	128.8 (4)	N13B—N1B—C14B—C15B	53.7 (5)
C2A—N1A—C14A—C15A	108.9 (5)	C2B—N1B—C14B—C19B	70.4 (5)
N13A—N1A—C14A—C15A	−53.3 (5)	N13B—N1B—C14B—C19B	−125.3 (4)
C19A—C14A—C15A—C16A	1.0 (7)	C19B—C14B—C15B—C16B	−2.9 (7)
N1A—C14A—C15A—C16A	−177.0 (4)	N1B—C14B—C15B—C16B	178.1 (4)
C14A—C15A—C16A—C17A	0.1 (7)	C14B—C15B—C16B—C17B	1.0 (7)
C15A—C16A—C17A—C18A	−1.6 (7)	C15B—C16B—C17B—C18B	1.3 (7)
C15A—C16A—C17A—Br1A	177.2 (3)	C15B—C16B—C17B—Br1B	−176.9 (3)
C16A—C17A—C18A—C19A	1.8 (7)	C16B—C17B—C18B—C19B	−1.6 (7)
Br1A—C17A—C18A—C19A	−177.0 (3)	Br1B—C17B—C18B—C19B	176.6 (3)
C15A—C14A—C19A—C18A	−0.7 (7)	C17B—C18B—C19B—C14B	−0.4 (7)
N1A—C14A—C19A—C18A	177.3 (4)	C17B—C18B—C19B—Cl1B	178.8 (3)
C15A—C14A—C19A—Cl1A	177.3 (3)	C15B—C14B—C19B—C18B	2.6 (7)
N1A—C14A—C19A—Cl1A	−4.8 (6)	N1B—C14B—C19B—C18B	−178.4 (4)
C17A—C18A—C19A—C14A	−0.7 (7)	C15B—C14B—C19B—Cl1B	−176.5 (3)
C17A—C18A—C19A—Cl1A	−178.7 (4)	N1B—C14B—C19B—Cl1B	2.4 (6)
N13A—C12A—C21A—C26A	−45.9 (6)	N13B—C12B—C21B—C26B	56.8 (6)
N11A—C12A—C21A—C26A	137.3 (4)	N11B—C12B—C21B—C26B	−125.1 (5)
N13A—C12A—C21A—C22A	130.9 (4)	N13B—C12B—C21B—C22B	−123.3 (5)
N11A—C12A—C21A—C22A	−45.9 (6)	N11B—C12B—C21B—C22B	54.8 (6)
C26A—C21A—C22A—C23A	−2.0 (6)	C26B—C21B—C22B—C23B	1.2 (7)
C12A—C21A—C22A—C23A	−178.9 (4)	C12B—C21B—C22B—C23B	−178.7 (4)
C21A—C22A—C23A—N24A	2.1 (7)	C21B—C22B—C23B—N24B	0.6 (8)
C22A—C23A—N24A—C25A	−0.8 (7)	C22B—C23B—N24B—C25B	−1.7 (8)
C23A—N24A—C25A—C26A	−0.6 (7)	C23B—N24B—C25B—C26B	1.1 (8)
C22A—C21A—C26A—C25A	0.7 (7)	C22B—C21B—C26B—C25B	−1.7 (7)
C12A—C21A—C26A—C25A	177.6 (4)	C12B—C21B—C26B—C25B	178.2 (4)
N24A—C25A—C26A—C21A	0.6 (7)	N24B—C25B—C26B—C21B	0.6 (8)
